# Efficacy and safety of 5 alpha-reductase inhibitor monotherapy in patients with benign prostatic hyperplasia: A meta-analysis

**DOI:** 10.1371/journal.pone.0203479

**Published:** 2018-10-03

**Authors:** Jae Heon Kim, Min Jung Baek, Hwa Yeon Sun, Bora Lee, Shufeng Li, Yash Khandwala, Francesco Del Giudice, Benjamin I. Chung

**Affiliations:** 1 Department of Urology, Stanford University Medical Center, Stanford, CA, United States of America; 2 Department of Urology, Soonchunhyang University Hospital, Soonchuhyang University Medical College, Seoul, Korea; 3 Department of Obstetrics and Gynecology, CHA Bundang Medical Center, Seongnam, Korea; 4 Department of Statistics, Graduate School of Chung-Ang University, Seoul, Korea; 5 Department of Urology and Dermatology, Stanford University Medical Center, Stanford, CA, United States of America; 6 University of California, San Diego School of Medicine, San Diego, CA, United States of America; 7 Department of Urology, Sapienza University of Rome, Rome, Italy; University Medical Center Utrecht, NETHERLANDS

## Abstract

**Background:**

Although combination therapy with 5 alpha-reductase inhibitor (5ARI) and alpha-blocker is one of the standard interventions in symptomatic benign prostatic hyperplasia (BPH), 5ARI monotherapy is seldom the focus of attention. Adverse events associated with 5ARI include depression and suicidal attempts in addition to persistent erectile dysfunction. The aim of this study is to update our knowledge of clinical efficacy and incidence of adverse events associated with 5ARI treatment in symptomatic BPH.

**Methods and findings:**

A meta-analysis of randomized controlled clinical trials (RCTs) from 1966 until March, 2017 was performed using database from PubMed, Cochrane Collaboration and Embase. A total of 23395 patients were included in this study and the inclusion criteria were: RCTs with 5ARI and placebo in symptomatic BPH patients. Parameters included prostate specific antigen (PSA), prostate volume (PV), International Prostate Symptom Score (IPPS), post-void residual urine (PVR), voiding symptoms of IPSS (voiding IPSS), maximum urinary flow rate (Qmax), and adverse events (AEs). A meta-analysis with meta-regression was performed for each effect size and adverse events, sensitivity analysis, cumulative analysis along with the analysis of ratio of means (ROM) in the placebo group.

A total of 42 studies were included in this study for review, and a total of 37 studies were included in the meta-analysis, including a total of 23395 patients (treatment group: 11392, placebo group: 12003). The effect size of all variables except PVR showed a significant improvement following 5ARI treatment compared with placebo. However, the effect size of differences showed declining trend in PV, IPSS and Qmax according to recent years of publication. In ROM analysis, PV showed no significant increase in the placebo group, with a ROM of 1.00 (95% CI, 0.88, 1.14). The 5ARI treatment resulted in a significantly higher incidence of decreased libido (OR = 1.7; 95% CI, 1.36, 2.13), ejaculatory disorder (OR = 2.94; 95% CI, 2.15, 4.03), gynecomastia (OR = 2.32; 95% CI, 1.41, 3.83), and impotence (OR = 1.74; 95% CI, 1.32, 2.29). Our study has the following limitations: included studies were heterogeneous and direct comparison of efficacy between alpha blocker and 5ARI was not performed. Adverse events including depression or suicidal attempt could not be analyzed in this meta-analysis setting.

**Conclusions:**

Although there was a significant clinical benefit of 5ARI monotherapy compared with placebo, the effective size was small. Moreover, the risk of adverse events including sexually related complications were high. Additional head-to-head studies are needed to re-evaluate the clinical efficacy of 5ARI compared with alpha-adrenergic receptor blockers.

## Introduction

Benign prostatic hyperplasia (BPH) with lower urinary tract symptoms (LUTS) is one of the most common diseases prevalent in old men. The prevalence of BPH among men in their 50s and 60s is 50% rising to 90% by the age of 80s and beyond based on autopsy findings [1, 2].

Medical treatment including alpha-blockers and 5 alpha-reductase inhibitors (5ARI) take possession of the primary treatment strategy in patients with BPH/LUTS [3, 4]. The combination of alpha-blockers and 5ARI improved LUTS and maximal urinary flow rate (Qmax)[2, 3]. In earlier 2000s, two important randomized controlled studies (RCTs) including the Medical Therapy of Prostatic Symptoms (MTOPS) [5] and the Combination of Avodart® and Tamsulosin (CombAT) study [6] established the superiority of long-term combination therapy over alpha-blocker monotherapy or placebo in the treatment of patients with BPH/LUTS. Furthermore, treatment using 5ARIs showed a positive effect including decreased prostate volume, improved International Prostate Symptom Score (IPSS), improved Qmax, decreased risk of acute urinary retention (AUR) and decreased operative procedures related with BPH/LUTS [5, 7–9]. Moreover, several systematic reviews showed that 5ARI, especially, finasteride improves LUTS by long-term treatment, however, combination treatment with alpha blockers showed better improvement than finasteride monotherapy [10, 11].

However, recent studies reported persistent complications of 5ARI including erectile dysfunction (ED) and decreased libido even after discontinuation of 5ARI [12–14]. Similarly, treatment with finasteride 1mg for androgenic alopecia has shown persistent ED after its withdrawal [15]. Currently, FDA recommends a change in 5ARI labeling to include the possibility of persistent adverse events even after discontinuation in several post-marketing studies [16, 17]. Two recent reviews of 5ARI are warning clinicians to inform their patients fully regarding the adverse events of erectile dysfunction, decreased libido, gynecomastia, and anxiety [18, 19]. Moreover, this 5ARI advisory was issued again about the possible risks for suicidal attempts and depression in many recent observational studies [20].

Evidence supports the efficacy of 5ARI treatment when combined with alpha-blockers. Several reviews and meta-analyses were limited to only adverse events. Therefore, we have provided an update on the clinical efficacy and adverse events in an effort to develop a rational therapeutic strategy using 5ARI in BPH/LUTS.

## Methods

The systematic review with meta-analysis and meta-regression were conducted according to the guidelines provided by the PRISMA guidelines (S1 Text).

### Inclusion criteria

This meta-analysis has inclusion criteria as randomized controlled clinical trials (RCTs) with 5ARI and placebo, disease indication of BPH/LUTS, and types of measure has to include at least one of followings: prostate specific antigen (PSA), prostate volume (PV), International Prostate Symptom Score (IPPS), post-void residual urine (PVR), voiding symptoms of IPSS (Voiding IPSS), maximum urinary flow rate (Qmax), and adverse events (AEs).

### Searching strategies

Based on the PICO (population, intervention, control, and outcomes) process, the following strategies were used: P (patients with symptomatic BPH); I (daily or regular maintenance treatment with 5ARI without any other treatment); C (comparing measured effect size with placebo group); and O (PSA, PV, IPSS, and Qmax). A MEDLINE search from 1966 to March 3, 2017 was performed using specific MeSH headings, including prostatic hyperplasia, lower urinary tract symptoms and 5 alpha-reductase inhibitors, dutasteride, and finasteride. Supplementary terms included dutasteride and finasteride. For natural headings, placebo, dutasteride and finasteride were included. A similar strategy was used for Cochrane collaboration and Embase (S2 Fig and S3 Fig). Detailed inclusion criteria for the final data extraction in the meta-analysis were: 1) reported outcomes of at least one of the variables included PSA, PV, IPSS, voiding IPSS, PVR, Qmax or adverse events; 2) daily 5ARI treatment; 3) indication for 5ARI use confined to BPH; 4) intention-to-treat analysis with placebo-controlled RCTs.

### Data extraction strategies

**Fig 1:**
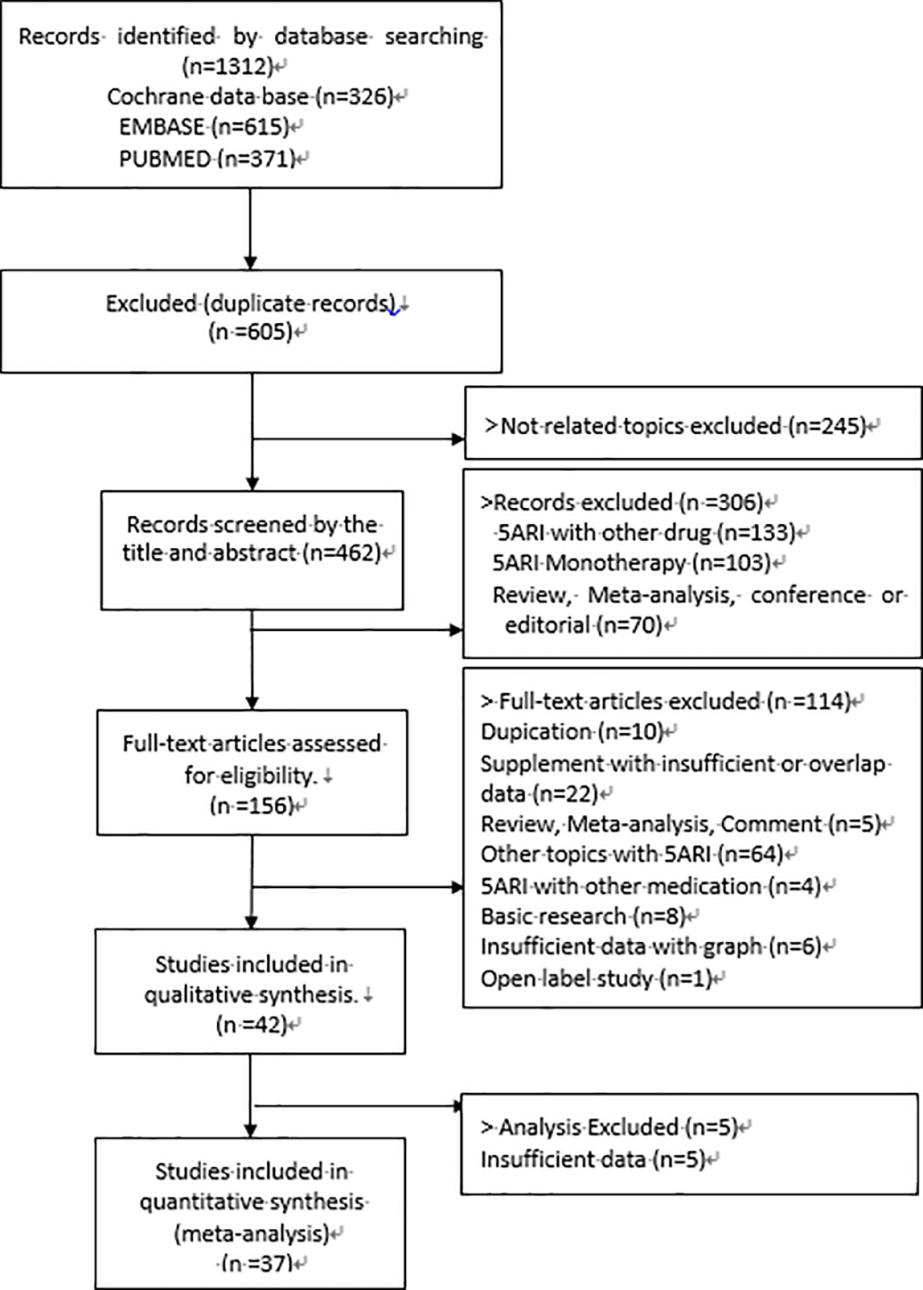
PRISMA flow diagram.

After merging all the search studies (n = 1312) from MEDLINE, Cochrane collaboration and Embase, duplicate studies (n = 605) were filtered (Fig 1). A total of 707 studies were screened by title, and a total of 245 studies involving unrelated topics were excluded. A total of 462 studies were screened by abstract and additionally, a total of 306 studies were excluded. Initial screening was performed by JHK and HYS. A total of 156 studies were reviewed for full text. Two authors (JHK and HYS) independently performed screening and full-text assessment, and all disagreements about final inclusion were reviewed by all authors. Data extraction was performed by independent fashion using standardized data extraction form.

### Assessment of methodological and reporting bias in included studies

Cochrane collaboration tools including random sequence generation, allocation concealment, blinding of participants and personnel, blinding of outcome assessment, incomplete outcome data, and selective reporting were used to assess the risk of methodological bias. Egger’s linear regression test was conducted to assess the publication bias.

### Statistical analysis

The effect of continuous outcomes was summarized as the standardized mean difference (SMD), which was estimated as the difference between the mean change in the treatment and placebo groups divided by the pooled standard deviation (SD). The unreported SDs were estimated from the reported ranges, p-values, standard errors, and sample sizes as described by Hozo, et al.[21] The SMD was interpreted as Cohen’s d: an SMD of 0.2 ~0.5 was considered small, an SMD of 0.5 to 0.8 moderate, and an SMD over 0.8 as a large effect [22].

The SMD exceeding 0.5 represented a clinically meaningful result. To identify the effect of placebo on the continuous outcomes, the ratio of means (ROM), which was a measure of relative change compared with the baseline, was used[23]. Binary outcomes were determined by estimating the odds ratio (OR) and 95% confidence interval (CI) using the Mantel-Haenszel methods.

To combine the results of individual studies, a meta-analysis was conducted based on the random effects model as described by DerSimonian and Laird using inverse variance weighting [24]. We pre-specified the type of medicine (finasteride, dutasteride, and 5ARI) as the stratified variable based on the assumption that the impact of the treatment varied by the type of medicine. The heterogeneity between studies was assessed for each outcome using I^2^ measure of inconsistency [25]. An I^2^ of 25–49% was interpreted as low heterogeneity, 50–74% was moderate, and high when it was greater than 75% [26]. Publication bias was examined by generating a funnel plot and performing the Egger’s asymmetry test.

Potential source of heterogeneity was investigated via cumulative meta-analysis (including sequential studies according to the publication year) and influential meta-analysis (deriving the pooled estimates by omitting one study at a time). The meta-regression analyses were performed using the publication year and follow-up duration to determine the related effect of the ROM in the placebo group.

Two-sided p value of <0.05 was considered as statistically significant and all the analyses were performed using R (version 3.4.1; The R Foundation for Statistical Computing, Vienna, Austria).

## Results

### Study inclusion

A total of 42 studies were included in this meta-analysis, involving a total of 37449 patients (treatment group: 18587, placebo group: 19162)(Table 1 and Fig 1). Five studies were excluded in the final analysis due to insufficient data. A total of 37 studies were finally included in the meta-analysis, involving a total of 23395 patients (treatment group: 11392, placebo group: 12003).

**Table 1:** Characteristics of all studies included in meta-analysis.

Publication			Country	No. of patients		Mean age(year)		Subject Description	Symptom of BPH			Experimental description		
Author	Journal	Year		Tx	Placebo	Tx.	Placebo		BPH	BOO	LUTS	Drug	dose (mg)	F/U duration (month)
Beisland	European Urology	1992	NA	94	88	60	60	Qmax<15mL/s	1	0	0	Finasteride	5	6
Gormley	The New England Journal of Medicine	1992	USA, Canada	297	300	64	64	Qmax<15mL/s, voided volume>150mL	1	0	0	Finasteride	5	12
Kirby	British Journal of Urology	1992	UK	31	21	64.4	64.4	Qmax<15mL/s, voided volume>150mL, detrusor pressure during voiding>50cmH2O2	1	1	0	Finasteride	5	3
Stoner	The Journal of Urology	1992	USA	18	25	63.9	63.9	Enlarged prostate gland of greater than 30cc	1	0	1	Finasteride	5	6
Tammela	The Journal of Urology	1993	Finland	19	17	65	65	Qmax<15mL/s, voided volume>150mL	1	1	0	Finasteride	5	6
Tempany	The Prostate	1993	USA	12	8	NA	NA	NA	1	0	0	Finasteride	1 or 5	12
The finasteride study group	The Prostate	1993	Australia, Belgium, Brazil, France, Italy, Mexico, Netherlands, New Zealand, Portugal, Spain, Swizerland, Germany, UK, USA	246	255	66	66	Qmax<15mL/s, PV>30cm^3^	1	0	1	Finasteride	5	12
Stoner	UROLOGY	1994	USA	291	299	64	64	Qmax<15mL/s, voided volume>150mL	1	0	0	Finasteride	5	12
			International study	242	254	66	66							
Andersen	UROLOGY	1995	Scandinavian countries(Denmark, Finland, Iceland, Norway, Sweden)	347	346	65.5	65.5	Qmax5-15mLs, PSA≤10ng/mL, PVR≤150cc	1	0	0	Finasteride	5	24
Tammela	The Journal of Urology	1995	Finland	12	15	65	65	Qmax<15mL/s, voided volume>150mL	1	1	0	Finasteride	5	6
Yu	Journal of the Formosan Medical Association	1995	Taiwan	24	22	66.4	65.2	NA	1	0	0	Finasteride	5	6
Lepor	The New England Journal of Medicine	1996	USA	305	310	65	65	AUA>8, Qmax4-15mL/s, voided volume>125mL	1	0	0	Finasteride	5	13
Nickel	Canadian Medical Association	1996	Canada	310	305	63	63.5	Qmax5-15mLs, voided volume>150mL, PVR≤150cc, PSA<10ng/mL	1	0	0	Finasteride	5	24
Habib	Clinical Endocrinology	1997	Scotland	19	9	68.7	66.7	NA	1	0	0	Finasteride	5	3
Lepor	The Journal of Urology	1998	USA	44	39	62.5	62.5	Qmax4-15mL/s, voided volume≥125mL, PVR≤300mL, AUA-SI score≥8	0	0	0	Finasteride	5	13
Marberger	UROLOGY	1998	USA	1450	1452	63	63.4	Qmax5-15mL/s, voided volume>150mL	1	0	0	Finasteride	5	24
McConnell	The New England Journal of Medicine	1998	USA	1524	1516	64	64	Qmax≤15mL/s, voided volume>150mL	1	0	0	Finasteride	5	48
Pannek	The Journal of Urology	1998	USA	26	14	65	64	IPSS>9, PSA≤10ng/mL	0	0	0	Finasteride	5	6
Abrams	The Journal of Urology	1999	USA	69	37	68.1	67.4	NA	0	0	0	Finasteride	5	9
Lukkarinen	Annales Chirurgiae et Gynaecologiae	1999	Finland	33	31	65	65	Boyarsky<15mL/s, PV>30cc	1	0	0	Finasteride	5	24
Schafer	UROLOGY	1999	Germany, Finland, UK, Sweden, Netherland, Denmark, Portugal, USA	81	40	68.1	NA	PSA<10ng/mL	1	1	0	Finasteride	5	12
Feneley	Prostate cancer and prostatic diseases	2000	UK, Netherland	18	9	67.5	67.5	BPH/BOO	1	1	0	Finasteride	NA	6
Isotalo	British Journal of Urology	2001	Finland	29	19	71	71	NA	1	0	1	Finasteride	5	18
Espana	BJU International	2002	Spain	30	10	66.7	69.5	Qmax≤15mL/s, IPSS>7, PVR<150mL, tPSA<20ng/mL	1	0	0	Finasteride	NA	9
Haggstrom	Scandinavian Journal of Urology and Nephrology	2002	Sweden	13	15	NA	NA	NA	0	0	0	Finasteride	5	3
Roehrborn	UROLOGY	2002	Global study	2167	2158	66.5	66.1	Qmax≤15mL/s, PSA≥1.5ng/mL, PV≥30cc, AUA-SI score≥12	1	0	0	Dutasteride	0.5	24
Kirby	UROLOGY	2003	Europe	239	253	63	64	Qmax5-15mLs, voided volume≥150mL, IPSS≥12, Prostate volume nearest 5g	1	0	0	Finasteride	5	13
McConnell	The New England Journal of Medicine	2003	NA	89	128	62.6	62.5	Qmax4-15mL/s, voided volume≥125mL, AUA-SI score 8–35	1	0	0	Finasteride	5	54
Roehrborn	The Journal of Urology	2004	USA	1524	1516	64	63.9	Qmax≤15mL/s, voided volume>150mL	1	0	0	Finasteride	5	48
Crawford	The Journal of Urology	2006	NA	NA	737	-	62.5	Qmax4-15mL/s, AUA-SI score>8, voided volume≥125mL	1	0	0	Finasteride	5	54
Gittelman	The Journal of Urology	2006	NA	2167	2158	65.8	65.5	Qmax>15mL/s, AUA_SI>12, PV>30cc, PSA1.5-10ng/mL	1	0	0	Dutasteride	0.5	48
Kaplan	The Journal of Urology	2006	USA	232	250	61	60.5	AUA8-35, Qmax4-15mL/s, voided volume>125mL, TPV<25	1	0	1	Finasteride	5	54
				281	274	61.8	62.4	AUA8-35, Qmax4-15mL/s, voided volume>125mL, TPV<40						
				252	213	65.1	64.8	AUA8-35, Qmax4-15mL/s, voided volume>125mL, TPV>40						
Kaplan	The Journal of Urology	2008	USA	768	737	62.6	62.5	Qmax 4-15mL/s, voided volume>125mL, AUA-SI score 8–30	0	0	0	Finasteride	5	54
Bepple	UROLOGY	2009	USA	30	29	66	66	NA	0	0	0	Dutasteride	0.5	12
Tsukamoto	Hinyokika Kiyo	2009	Japan	70	70	66.1	65.8	Qmax>15mL/s, IPSS>3	0	0	0	Dutasteride	0.5	6
Tsukamoto	International Journal of Urology	2009	Japan	193	185	67.7	64.4	Qmax<15mLs, IPSS≥8, voided volume≥150mL, PV>30mL	1	0	0	Dutasteride	0.5	13
Tsukamoto	Hinyokika Kiyo	2010	Japan	184	181	68	66.9	Qmax<15mLs, IPSS≥8, PV<30cc	1	0	0	Dutasteride	0.5	13
Kaplan	The Journal of Urology	2011	USA	281	276	60.7	60.3	Qmax 4-15mL/s, AUA-SI score 8–30 voided volume>125mL, PV<30mL	0	0	0	Finasteride	5	54
				295	288	63.9	64.1	Qmax4-15mL/s, AUA-SI score 8–30 voided volume>125mL, PV>30mL						
Roehrborn	UROLOGY	2011	USA	4049	4073	62.7	62.7	50–60 years old: PSA2.5-10ng/mL, >60 years old: 3.0-10ng/mL	1	0	0	Dutasteride	0.5	48
Yanqun	Clinical Drug Investigation	2012	China	126	127	65.8	66.9	Qmax 5-15mL/s, AUA-SI score>12 voided volume>125mL,	1	0	0	Dutasteride	0.5	6
Kacker	Androlodia	2015	USA	11	11	57.7	57.7	Testosterone (T) for at least 3 months, and a current serum T within the normal range (300–1000ng dl)	0	0	0	Dutasteride	0.5	12
Qian	The Aging Male	2015	China	45	42	70.1	72.3	PV>80mL, IPSS≥13, QoL≥3, PVR200mL, Qmax<15mL/s, refractory HU history, bladder stone history, AUR history, refractory UTI history	1	0	0	5ARI Finasteride or Dutasteride	5 or 0.5	6

NA, not available; BPH, benign prostatic hyperplasia; 5ARI, 5 alpha reductase inhibitor; PVR, post voided residual volume; PSA, prostatic specific antigen; PV, prostatic volume; IPSS, International Prostate Symptom Score; Qmax, maximal urinary flow rate; QoL, quality of life. UTI, urinary tract infection

### Risk of bias

For random sequence generation, most of the included studies showed a low risk and only a single study showed unclear risk (S1 Table). For allocation concealment, 28 studies showed unclear risk and 14 studies showed low risk. For blinding of participants and personnel (performance bias), 7 studies showed high risk, one study showed unclear risk and 34 studies showed low risk.

### Effective size of PSA, PV, IPSS, and Qmax of 5ARI compared with placebo

**Fig 2:**
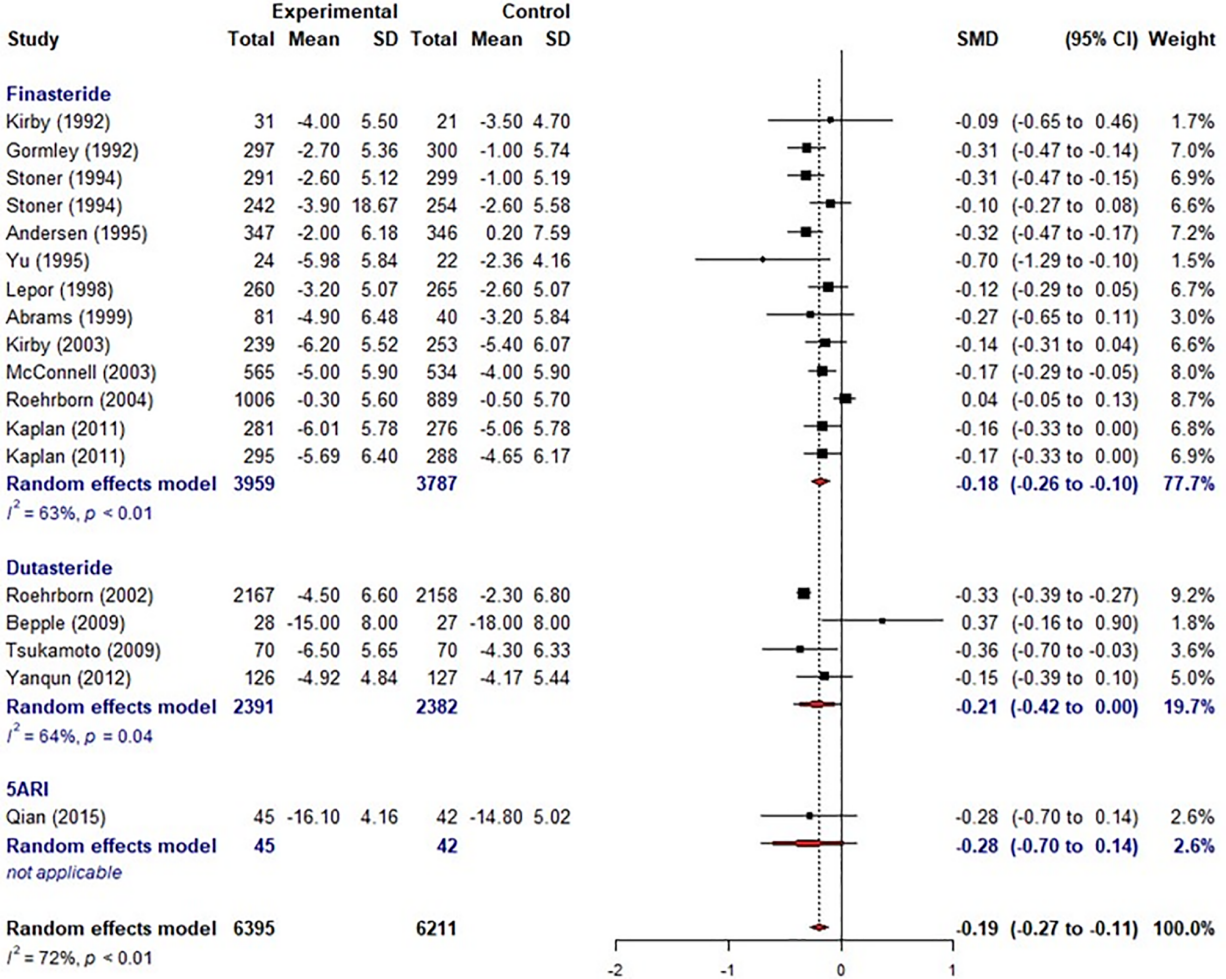
Meta-analysis of effective sizes in prostate specific antigen (PSA), International Prostate Symptom Score (IPSS), prostate volume and maxima urinary flow rate (Qmax).

For PSA, a total of 13 studies were included in the meta-analysis. The overall effective size based on SMD was -0.76 (95% CI, -1.31, -0.22)(S1 Fig), which showed a significant decrease in PSA level after 5ARI treatment compared with placebo. The overall effective size showed moderate effect of improvement by Cohen’s cutoff. The overall effective size based on WMD was -1.27 (-2.29, -0.24). For PV, a total of 26 studies were included in the meta-analysis. The overall effective size based on SMD was -0.63 (95% CI, -0.74, -0.52)(S2 Fig), which showed a significant decrease of PV after 5ARI medication compared with placebo. The overall effective size showed moderate effect of improvement by Cohen’s cutoff. The overall effective size based on WMD was -11.13 (-13.34, -8.93). For PVR, a total of 5 studies were included in the meta-analysis. The overall effective size by SMD was 0.1 (95% CI, -0.48, 0.68), which showed insignificant decrease of PVR after 5ARI treatment compared with placebo. For IPSS, a total of 18 studies were included in the meta-analysis. The overall effective size by SMD was -0.19 (95% CI, -0.27, -0.11)(Fig 2), which showed a significant decrease of IPSS after 5ARI intervention compared with placebo. The overall effective size showed small effect of improvement by Cohen’s cutoff. The overall effective size based on WMD was -1.21(-1.72, -0.70). Finasteride showed a significant improvement of IPSS as -0.18 (95% CI, -0.26, -0.10). However, dutasteride showed no significant improvement in IPSS as -0.21 (95% CI, -0.42, 0.00). For Qmax, a total of 23 studies were included in the meta-analysis, and the overall effective size by SMD was 0.29 (95% CI, 0.22 to 0.36)(S3 Fig), which showed significant improvement of Qmax after 5ARI treatment compared with placebo. The overall effective size based on WMD was -1.16 (0.88, 1.43).

### Cumulative analysis

**Fig 3:**
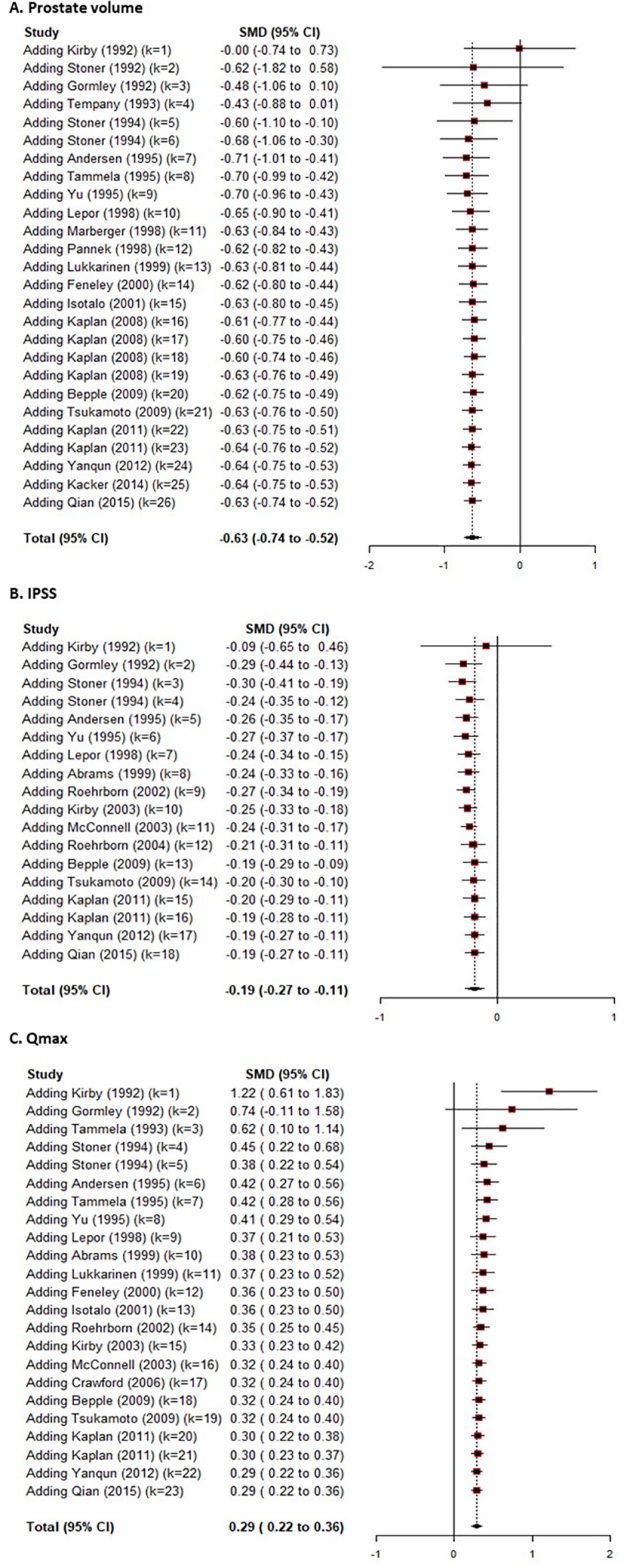
Cumulative analysis of effective sizes in prostate volume, International Prostate Symptom Score (IPSS), and maxima urinary flow rate (Qmax).

Cumulative meta-analysis was performed to investigate the trend according to years. For PSA, in 1990s, effective size showed no significant difference compared with placebo. However, starting with 2000s, the effective size showed a significant difference compared with placebo, resulting in a stable outcome from -0.77 to -0.60. For PV, the effect size showed a constant and significant difference compared with placebo, which showed stable outcome from -0.70 to -0.40 (Fig 3A). For IPSS, there was no marked change in the trend of effective size compared with placebo, however, the effective size of difference compared with placebo showed a decreasing trend (Fig 3B). For Qmax, the effective size showed a large difference compared with placebo until 1993. However, from 1994, the effective size of difference showed a decreasing trend as a convergence of 0.3 (Fig 3C).

### Sensitivity analysis

Considering the relatively high heterogeneity, sensitivity analysis was performed to analyze the effect of each study. Overall effect size of all variables except PVR showed no impact of individual studies (S4 Fig). However, the effect size of PVR was affected by one study, which resulted in a different outcome without that study.

### Meta-regression

To investigate the reasons for heterogeneity of effect, a meta-regression analysis was performed. Moderating factor was suggested as IPSS at baseline, follow-up duration and race. Effective sizes of all variables except PVR showed no significant moderating effect. However, the effective size of PVR was affected by IPSS at baseline. The effective size of PVR decreased according to high IPSS at the baseline: -0.29 (95% CI, -0.46,-0.12) in univariate analysis. Multivariable analysis was not performed due to the small number of included studies.

### ROM analysis of placebo group

To show the placebo effect of each variable, a ROM analysis was performed (Table 2). For PSA, although it did not show a significant effective size, it still showed a decrease by 10% (0.90 (95% CI, 0.81, 1.00) during follow-up. For PV, there was no change during follow-up: 1.00 (95% CI, 0.88, 1.14). For IPSS, there was a significant decrease during follow-up: 0.77 (95% CI, 0.68, 0.88), suggesting a 23% improvement. For Qmax, there was significant increase during the follow-up: -1.13 (95% CI, 1.06, 1.20), suggesting a 13% aggravation.

**Table 2:** Ratio of means meta-analysis of the efficacy of placebo group.

Author (year)	No. of samples	Ratio of mean (95% CI)^a^					
		PSA	PV	PVR	IPSS	voiding IPSS	Qmax
Gormley (1992)	300		0.98 (0.89, 1.08)		0.90 (0.81, 0.99)	0.88 (0.80, 0.97)	1.02 (0.96, 1.08)
Kirby (1992)	10	0.80 (0.39, 1.64)	0.96 (0.49, 1.87)	0.52 (0.16, 1.64)	0.81 (0.48, 1.39)		0.90 (0.54, 1.51)
Tempany (1993)	8		1.01 (0.63, 1.62)				
Tammela (1993)	17			1.10 (0.64, 1.88)			1.12 (0.90, 1.41)
Andersen (1995)	197		1.28 (1.04, 1.56)		1.02 (0.93, 1.11)	0.98 (0.90, 1.06)	0.97 (0.93, 1.02)
Tammela (1995)	15		0.96 (0.72, 1.27)	1.19 (0.63, 2.24)			1.11 (0.87, 1.42)
Yu (1995)	22	0.89 (0.51, 1.58)	1.06 (0.83, 1.37)		0.86 (0.73, 1.01)		1.01 (0.80, 1.29)
Habib (1997)		1.26 (0.77, 2.05)					
Lepor (1998)	258		1.01 (0.94, 1.09)		0.84 (0.79, 0.89)		1.13 (1.08, 1.20)
Marberger (1998)	800		1.04 (0.99, 1.09)				
Pannek (1998)	10	0.73 (0.28, 1.88)	0.97 (0.69, 1.37)				
Abrams (1999)					0.82 (0.69, 0.96)		0.99 (0.88, 1.11)
Lukkarinen (1999)	31		0.92 (0.76, 1.10)				0.84 (0.67, 1.06)
Feneley (2000)	9		0.82 (0.52, 1.31)				1.23 (0.85, 1.77)
Isotalo (2001)	19	0.66 (0.53, 0.81)	0.91 (0.75, 1.11)				1.09 (0.76, 1.56)
Haggstrom (2002)		1.02 (0.86, 1.21)					
Kirby (2003)		1.12 (0.96, 1.29)			0.69 (0.63, 0.74)		1.12 (1.06, 1.18)
McConnell (2003)					0.76 (0.73, 0.80)		1.13 (1.10, 1.16)
Roehrborn (2004)		0.89 (0.84, 0.95)			0.97 (0.94, 1.00)		
Crawford (2006)							1.13 (1.11, 1.16)
Kaplan (2008)	249		1.34 (1.22, 1.46)				
Kaplan (2008)a	214		1.12 (1.04, 1.21)				
Kaplan (2008)b	112		1.20 (1.08, 1.32)				
Kaplan (2008)c	161		1.21 (1.16, 1.27)				
Tsukamoto (2009)	70		0.81 (0.69, 0.95)		0.73 (0.62, 0.85)		1.12 (0.99, 1.27)
Qian (2015)	42	0.81 (0.77, 0.86)	0.60 (0.57, 0.63)	0.06 (0.05, 0.07)	0.36 (0.32, 0.41)		2.79 (2.36, 3.30)
Overall		0.90 (0.81, 1.00)	1.00 (0.88, 1.14)	0.44 (0.06, 3.22)	0.77 (0.68, 0.88)	0.93 (0.84, 1.03)	1.13 (1.06, 1.20)
		p = 0.056	p = 0.97	p = 0.42	p<0.001	p = 0.159	p<0.001
Heterogeneity—I^2^ (%)		73.3 (47.8, 86.3)	96.3 (95.1, 97.1)	98.5 (97.6, 99.0)	96.7 (95.4, 97.6)	61.9 (0.0, 91.2)	91.3 (87.5, 93.9)
p-value		<0.001	<0.001	<0.001	<0.001	0.105	<0.001

CI, confidence interval; PSA, prostate specific antigen; PV, prostate volume; PVR, post voided residual volume; IPSS, International Prostate Symptom Score; Qmax, maximal urinary flow rate.

^a^ The process of meta-analysis with paired difference data: estimates using the Hegde's corrected standardized mean difference assuming the random-effect model

### Meta-regression of ROM analysis in placebo group

To investigate the reasons for the placebo effect in ROM analysis, the published year and follow-up duration were suggested as moderators (S2 Table). For PSA, there was no significant moderator effect. However, for PV, IPSS, and Qmax, both published year and follow-up duration were significant moderators. The effective size of PV and IPSS was decreased according to years of recent publication (p<0.001), and was increased according to follow-up duration (p<0.001). In contrast, the effective size of Qmax was increased according to years of recent publication (p<0.001), and decreased by the follow-up duration (p = 0.020). For PVR, only the year of publication affected the effective size, which showed a decreased effect according to the year of recent publication (p<0.001).

### Adverse events

The 5ARI treatment prevented exacerbation of BPH and urinary retention compared with placebo (Table 3). However, compared with placebo, 5ARI showed a significantly higher incidence of decreased libido (OR = 1.7; 95% CI, 1.36, 2.13), ejaculatory disorder (OR = 2.94; 95% CI, 2.15, 4.03), gynecomastia (OR = 2.32; 95% CI, 1.41, 3.83), and impotence (OR = 1.74; 95% CI, 1.32, 2.29). Decreased libido and impotence was affected by the moderating effect of follow-up duration. After meta-regression of follow up duration, decreased libido (OR = 0.98, 95% CI, 0.97, 0.99) and increased impotence (OR = 0.98, 95% CI, 0.97, 0.99) were significantly related to longer follow-up duration.

**Table 3:** Meta-analysis and meta-regression of effective sizes in adverse events.

Complication	Effect size			Meta-regression on f/u duration (month)	
	OR (95% CI)	p-value	I^2^	OR (95% CI)	p-value
Abdominal pain	1.16 (0.78–1.74)	0.455	0.0%	0.97 (0.91–1.04)	0.377
Gormley (1992)	3.37 (0.32–35.31)	0.311			
Marberger (1998)	1.06 (0.68–1.67)	0.784			
Stoner (1994)	1.7 (0.41–7.09)	0.464			
Tsukamoto (2009)	0.14 (0.01–2.62)	0.186			
Tsukamoto (2009)	2.03 (0.53–7.8)	0.303			
Angina pectoris	1.01 (0.55–1.84)	0.977	NA%	NA	NA
Marberger (1998)	1.01 (0.55–1.84)	0.977			
Any AE	0.98 (0.88–1.09)	0.767	56.8%	1.01 (0.99–1.03)	0.368
Andersen (1995)	1.3 (0.83–2.05)	0.251			
Beisland (1992)	1 (0.75–1.33)	0.992			
Kacker (2015)	NA (NA-NA)	NA			
Nickel (1996)	1 (0.93–1.08)	0.922			
The finasteride study group (1993)	12.44 (1.63–94.95)	0.015			
Tsukamoto (2009)	0.98 (0.91–1.05)	0.505			
Tsukamoto (2009)	0.83 (0.67–1.03)	0.09			
Asthenia	0.86 (0.59–1.25)	0.43	2.5%	0.93 (0.87–1)	0.046
Gormley (1992)	1.01 (0.21–4.96)	0.99			
Kirby (2003)	1.02 (0.45–2.31)	0.964			
Lepor (1996)	1.08 (0.61–1.91)	0.797			
Marberger (1998)	0.46 (0.23–0.94)	0.033			
Stoner (1994)	1.28 (0.34–4.73)	0.714			
Back pain	0.61 (0.39–0.95)	0.028	0.0%	NA	NA
Marberger (1998)	0.59 (0.37–0.95)	0.029			
Tsukamoto (2009)	0.76 (0.18–3.28)	0.713			
BPH worsening	0.55 (0.37–0.83)	0.004	NA%	NA	NA
Marberger (1998)	0.55 (0.37–0.83)	0.004			
Breast pain	2.49 (0.88–7.01)	0.084	0.0%	NA	NA
Gormley (1992)	3.03 (0.12–74.09)	0.497			
McConnell (1998)	2.43 (0.81–7.26)	0.112			
Bronchitis	1.15 (0.7–1.91)	0.579	NA%	NA	NA
Marberger (1998)	1.15 (0.7–1.91)	0.579			
Decreased libido	1.67 (1.35–2.06)	<0.001	9.8%	0.98 (0.97–0.99)	0.004
Bepple (2009)	6.53 (0.35–120.66)	0.208			
Gormley (1992)	3.63 (1.19–11.01)	0.023			
Kirby (2003)	1.83 (0.62–5.4)	0.271			
Lepor (1996)	3.44 (1.15–10.34)	0.028			
Marberger (1998)	1.44 (0.99–2.11)	0.057			
McConnell (1998)	1.01 (0.65–1.56)	0.965			
Nickel (1996)	1.59 (0.92–2.76)	0.095			
Roehrborn (2002)	1.97 (1.39–2.79)	<0.001			
Stoner (1994)	2.04 (0.93–4.51)	0.077			
Tsukamoto (2009)	4.77 (0.23–98.64)	0.312			
Yanqun (2012)	2.02 (0.19–21.95)	0.565			
Yu (1995)	4.59 (0.23–90.58)	0.316			
Diarrhea	1.05 (0.58–1.9)	0.863	0.0%	NA	NA
Gormley (1992)	NA (NA-NA)	NA			
Marberger (1998)	1.15 (0.56–2.35)	0.696			
Tsukamoto (2009)	0.87 (0.31–2.46)	0.792			
Dizziness	1.06 (0.74–1.52)	0.754	0.0%	1.09 (0.88–1.34)	0.451
Gormley (1992)	0.19 (0.01–3.99)	0.288			
Kirby (2003)	1.07 (0.59–1.93)	0.822			
Lepor (1996)	1.16 (0.67–2.01)	0.588			
Stoner (1994)	1.02 (0.26–4.07)	0.975			
Tsukamoto (2009)	4.77 (0.23–98.64)	0.312			
Tsukamoto (2009)	0.68 (0.2–2.29)	0.53			
Dyspepsia	0.34 (0.01–8.16)	0.504	NA%	NA	NA
Tsukamoto (2009)	0.34 (0.01–8.16)	0.504			
Dysuria	1.34 (0.66–2.72)	0.417	0.0%	NA	NA
Gormley (1992)	5.05 (0.24–104.75)	0.295			
Marberger (1998)	1.24 (0.6–2.57)	0.56			
Ejaculatory disorder	2.89 (2.12–3.93)	<0.001	0.0%	1 (0.98–1.03)	0.689
Gormley (1992)	2.57 (0.94–7.08)	0.067			
Kirby (2003)	1.53 (0.44–5.35)	0.507			
Lepor (1996)	1.48 (0.42–5.18)	0.543			
Marberger (1998)	3.7 (1.78–7.7)	<0.001			
McConnell (1998)	3.01 (1.33–6.81)	0.008			
McConnell (1998)	1.99 (0.28–14.11)	0.491			
Nickel (1996)	4.69 (1.81–12.14)	0.001			
Roehrborn (2002)	2.81 (1.62–4.87)	<0.001			
Tsukamoto (2009)	4.06 (0.46–35.41)	0.205			
Erectile dysfunction	1.51 (0.15–15.27)	0.725	34.1%	NA	NA
Tsukamoto (2009)	3.81 (0.43–33.8)	0.229			
Yanqun (2012)	0.34 (0.01–8.17)	0.503			
Flatulence	1.39 (0.45–4.34)	0.568	0.0%	NA	NA
Gormley (1992)	1.44 (0.25–8.36)	0.682			
Stoner (1994)	1.36 (0.31–6.04)	0.687			
Gastritis	1.3 (0.65–2.6)	0.463	NA%	NA	NA
Marberger (1998)	1.3 (0.65–2.6)	0.463			
Gynecomastia	2.29 (1.4–3.76)	0.001	19.9%	0.98 (0.95–1.01)	0.119
McConnell (1998)	1.61 (0.88–2.95)	0.124			
Roehrborn (2002)	3.11 (1.78–5.45)	<0.001			
Yanqun (2012)	3.02 (0.12–73.53)	0.497			
Headache	0.97 (0.62–1.52)	0.906	38.0%	0.96 (0.91–1.01)	0.137
Beisland (1992)	1.4 (0.52–3.78)	0.502			
Gormley (1992)	0.96 (0.14–6.63)	0.969			
Lepor (1996)	1.87 (0.88–3.95)	0.102			
Marberger (1998)	0.92 (0.58–1.48)	0.743			
Marberger (1998)	0.42 (0.2–0.88)	0.021			
Stoner (1994)	1.7 (0.41–7.09)	0.464			
Tsukamoto (2009)	0.51 (0.1–2.68)	0.424			
Hypertension	0.82 (0.58–1.14)	0.239	0.0%	NA	NA
Kirby (2003)	0.75 (0.35–1.6)	0.452			
Marberger (1998)	0.83 (0.57–1.22)	0.347			
Hypotension	0.51 (0.09–2.76)	0.434	NA%	NA	NA
Kirby (2003)	0.51 (0.09–2.76)	0.434			
Impotence	1.68 (1.3–2.17)	<0.001	58.1%	0.98 (0.97–0.99)	0.001
Gormley (1992)	1.98 (0.69–5.68)	0.204			
Kirby (2003)	1.47 (0.64–3.38)	0.363			
Lepor (1996)	2.04 (1.1–3.78)	0.024			
Marberger (1998)	1.42 (1.06–1.89)	0.018			
McConnell (1998)	1 (0.74–1.36)	0.981			
Nickel (1996)	2.52 (1.52–4.18)	<0.001			
Roehrborn (2002)	1.83 (1.42–2.36)	<0.001			
Stoner (1994)	3.07 (1.31–7.15)	0.01			
Influenza	0.89 (0.58–1.37)	0.602	NA%	NA	NA
Marberger (1998)	0.89 (0.58–1.37)	0.602			
Lens change	1.2 (0.3–4.81)	0.793	12.7%	NA	NA
Gormley (1992)	5.05 (0.24–104.75)	0.295			
Gormley (1992)	0.19 (0.01–3.99)	0.288			
Stoner (1994)	1.36 (0.31–6.06)	0.684			
Myocardial infarction	2.9 (1.3–6.46)	0.009	NA%	NA	NA
Marberger (1998)	2.9 (1.3–6.46)	0.009			
Nausea	0.73 (0.23–2.28)	0.582	0.0%	NA	NA
Gormley (1992)	0.67 (0.11–4)	0.664			
Stoner (1994)	0.76 (0.17–3.4)	0.724			
orgasm dysfunction	0.8 (0.08–8.3)	0.85	31.3%	NA	NA
Gormley (1992)	2.24 (0.19–26.87)	0.523			
Stoner (1994)	0.2 (0.01–4.25)	0.305			
Pelvic pain	0.48 (0.04–5.18)	0.546	NA%	NA	NA
Gormley (1992)	0.48 (0.04–5.18)	0.546			
Pharyngitis	1.6 (0.78–3.28)	0.202	NA%	NA	NA
Marberger (1998)	1.6 (0.78–3.28)	0.202			
Postural hypotension	1.18 (0.27–5.12)	0.821	46.5%	NA	NA
Kirby (2003)	0.51 (0.09–2.76)	0.434			
Lepor (1996)	2.3 (0.6–8.8)	0.225			
Rash	1.59 (0.63–4.01)	0.326	38.4%	1.03 (0.97–1.09)	0.39
Gormley (1992)	2.24 (0.19–26.87)	0.523			
Marberger (1998)	0.82 (0.43–1.54)	0.532			
McConnell (1998)	5.31 (0.93–30.3)	0.061			
Stoner (1994)	2.04 (0.38–11.11)	0.408			
Rhinitis	0.56 (0.24–1.32)	0.186	NA%	NA	NA
Lepor (1996)	0.56 (0.24–1.32)	0.186			
Sinusitis	0.98 (0.25–3.9)	0.982	NA%	NA	NA
Lepor (1996)	0.98 (0.25–3.9)	0.982			
Somnolence	1.36 (0.48–3.86)	0.565	NA%	NA	NA
Kirby (2003)	1.36 (0.48–3.86)	0.565			
Syncope	1.63 (0.08–31.47)	0.747	46.0%	NA	NA
Kirby (2003)	0.34 (0.01–8.3)	0.508			
Lepor (1996)	6.89 (0.36–132.77)	0.201			
Testicular pain	1.4 (0.45–4.35)	0.565	0.0%	NA	NA
Gormley (1992)	1.44 (0.25–8.36)	0.682			
Stoner (1994)	1.36 (0.31–6.06)	0.684			
upper respiratory infection	0.7 (0.41–1.19)	0.191	NA%	NA	NA
Marberger (1998)	0.7 (0.41–1.19)	0.191			
urinary retention	0.49 (0.28–0.87)	0.015	NA%	NA	NA
Marberger (1998)	0.49 (0.28–0.87)	0.015			
Urinary tract Infection	0.71 (0.44–1.14)	0.154	NA%	NA	NA
Marberger (1998)	0.71 (0.44–1.14)	0.154			
Vertigo	2.04 (0.52–8.06)	0.31	NA%	NA	NA
Kirby (2003)	2.04 (0.52–8.06)	0.31			

OR, odds ratio; CI, confidence interval.

### Publication bias

There was no publication bias detected following Egger’s test (S5 Fig).

## Discussion

Although current guidelines suggest the use of 5ARI in patients with prostate size greater than 30cc, our study does not support the wide use of 5ARI, rather it needs specific indication. Although there has been a systematic review about this issue, especially for finasteride [11], it needs to be upgraded. The main academic basic hypothesis of our study is based on the most recent focus on androgens in aged men. It is well known that androgens profoundly regulate prostate growth and differentiation, as well as sexual function [12, 18]. However, it is also associated with general health of aged men including cardiovascular disease. A recent RCT showed that a 1-year treatment of testosterone showed superior outcomes compared with placebo in coronary artery non-calcified plaque volume [27]. In the Reduction by Dutasteride of Prostate Cancer Events (REDUCE) trial, the dutasteride treatment group showed larger rate of cardiac failure compared with placebo [28]. Moreover, the 5ARI treatment was again associated with the possible risk of suicidal attempts and depression in many observational studies [20].

It is beyond dispute that the combination treatment of alpha-blocker and 5ARI is superior to alpha-blocker monotherapy or placebo group [5, 6]. In early trials, the effect of combination treatment was mainly attributed to alpha-blocker and also to the double-placebo effect. In MTOPS trial, the overall effect of two placebo combinations was -4.0 at 1 year and also -4.0 at 4 years, which suggests a 23.8% improvement in placebo effect [5]. Further, at 1 year, there was no significant difference in symptom improvement between the alpha-blocker and combination groups [5]. Due to the absence of meta-analysis involving alpha-blocker, 5ARI, and placebo, this study failed to confirm the poor clinical efficacy of 5ARI compared with alpha-blockers. However, this is the first meta-analysis investigating the clinical efficacy of 5ARI monotherapy among RCTs, which showed that overall effective size of IPSS improvement by 5ARI compared with placebo was small. Moreover, the overall effective size of PV and PSA was moderate.

For PV growth, previous studies reported an annual growth rate of 0.6 cc per year (−9.9~11.8) [29]. Interestingly, the negative PV growth rate represents the diversity of growth rate according to individual characteristics. Loeb et al [30] reported that a considerable proportion of aging men do not show progressive PV enlargement, and a few manifested decreasing pattern. In their study of median follow up of 4.3 years, a progressive PV growth was noted in 61.9%. However, 38.1% of men showed no increase or decrease in PV with the rate of PV changing by 0.6cc annually (-9.9~62.1). They speculated that aging could induce prostate shrinkage in healthy community men due to genetic, hormonal or environmental factors. In another study of Olmsted county survey, the median growth rate of PV was 1.9% per year [31]. In our study, it was not logical to estimate the annual grow rate because this study does not contain direct population data. However, this study demonstrated the changes of PV in placebo group, which suggested that the PV growth rate was 1.00 by ROM (95% CI, 0.88–1.14), which showed lack of increase in PV size during the years of investigation of enrolled studies.

For adverse events, two recent reviews reported adverse effects including ED, decreased libido, gynecomastia, and anxiety following 5ARI therapy [18, 19]. Our study also found similar adverse effects including decreased libido, ejaculatory disorder, gynecomastia and impotence. Interestingly, Corona et al [18] reported that adverse events were inversely correlated with study duration. Our study also showed similar findings of decreased libido and impotence affected by the moderating effect of follow-up duration, which showed attenuation of decreased libido and impotence during a longer follow-up. Further studies are needed by merging data of 5ARI studies with other indications such as alopecia. Moreover, comorbidity status should be taken into account when considering the incidence of ED in patients receiving 5-ARIs. 5-ARIs are often prescribed to older patients with high comorbidity status, which could also increase the risk of ED [32]. The comorbidity status is often underreported in published studies and this may represent a bias.

The detailed mechanism underlying the negative impact of 5ARI on ED or decreased libido has not been fully demonstrated. Several suggested theories include: 1) Decrease synthesis of neurostransmitters by 5ARI [13], which is related to sexual desire; 2) 5ARIs induce structural and functional degeneration of penile tissue, which results in penile fibrosis due to cholinergic and nitrergic sensitivity [13]. In animal studies, finasteride suppressed neurosteroid synthesis, which resulted in anxiety and depression [33–35], which could explain the possible association between 5ARI treatment and depression or suicidal attempts.

Our study is academically sound and robust due to several reasons: 1) It is the first scientific review including meta-analysis of RCTs investigating the efficacy and adverse events associated with 5ARI monotherapy; 2) It shows an indirect effect of the reported years using a cumulative meta-analysis; 3) It shows indirect outcomes of prostate growth using a single-placebo-controlled meta-analysis. In our study, although the effective size of PSA, IPSS, PV, and Qmax showed significantly superior outcome compared with the placebo group, the effective size, especially for IPSS (-0.19, 95%CI: -0.27- -0.11) was small. Considering the effective size was analyzed by SMD, it could be interpreted as 7.5% by two sided test and as 15% by one sided test. By subgroups, although finasteride showed a significant improvement of IPSS, dutasteride showed not significant improvement, which is due to relatively lower published studies of dutasteride than finasteride. The included studies showed a lower clinical improvement in IPSS, PV and Qmax when recent publications were considered. PV showed a relatively slower growth than in previous clinical studies.

Due to limitation of meta-analysis, detailed individual level data could not be extracted. Furthermore, we could not find a long term complication including depression and suicidal attack, which could not be reported in ITT study design. For those complications, observational studies have to be included. This study also included outdated studies which only have focused on finasteride, which could result in favorable effect of finasteride than dutasteride during meta-analysis. One more crucial limitation in our study is that several studies among final included studies for meta-analysis do not have clinically significant BOO, which could resulted in lowering efficacy of 5ARI during meta-analysis. Moreover, recent meta-analysis about the efficacy of 5ARI in BOO has showed that BOOI reduction is important clinical outcomes during BPH/LUTS treatment [10]. Lastly, network analysis among alpha blocker, 5ARI, and placebo are needed to suggest the direct evidence of inferior clinical efficacy of 5ARI compared to alpha blocker.

In future, the accurate prevalence rate of persistent adverse events after 5ARI discontinuation needs to be investigated. Moreover, considering the long-term clinical efficacy of 5ARI, long-term adverse events need to be investigated more clearly. This meta-analysis provides useful information for clinicians and clinical investigators to design controlled studies investigating long-term outcomes following 5ARI therapy.

## Conclusions

In this meta-analysis with an average follow-up duration of 21.8 months, the efficacy outcomes of 5ARI showed a small clinical improvement in improvement of LUTS. In future, well designed studies are needed to overcome placebo effect and heterogeneities and possible bias. Considering persistent and well known adverse events including ED and decreased libido even after discontinuation of 5ARI, 5ARI therapy should be prescribed with great caution and patients need to be fully informed about the possible adverse events. A more selective rationale is needed considering the diverse growth rate of PV, and a relatively low growth rate observed in our study.

## Supporting information

S1 Text PRISMA checklist. (DOC)

S2 Text Searching strategies using Pubmed database. (PDF)

S3 Text Searching strategies using Cochrane database. (PDF)

S1 Table Methodological qualities of included studies. (DOCX)

S2 Table Meta-regression analysis for ratio of means meta-analysis of the efficacy of placebo group. (DOCX)

S1 Fig Meta-analysis of effective sizes in prostate specific antigen (PSA). (JPG)

S2 Fig Meta-analysis of effective sizes in prostate volume. (TIF)

S3 Fig Meta-analysis of maxima urinary flow rate (Qmax). (TIF)

S4 Fig Sensitivity analysis of effective sizes in prostate volume, International Prostate Symptom Score (IPSS), and maxima urinary flow rate (Qmax). (TIF)

S5 Fig Funnel plots for PSA, PV, IPSS, and Qmax. (JPG)
