# Supplementary material for: Efficacy and safety of 5 alpha-reductase inhibitor monotherapy in patients with benign prostatic hyperplasia: A meta-analysis
Source: PLoS One. 2018 Oct 3;13(10):e0203479. doi: 10.1371/journal.pone.0203479 (PMC6169865; doi:10.1371/journal.pone.0203479)
Supplement: S2 Text — (PDF) [file pone.0203479.s002.pdf]

Search	Add to builder	Query	Items found
#47	Add	Search **#37 NOT #40** Filters: **Publication date from 2015/01/01 to 2017/12/31**	31
#46	Add	Search **#37 NOT #40**	371
#40	Add	Search **#38 OR #39**	2227158
#39	Add	Search **"review literature as topic"[MeSH Terms]**	9049
#38	Add	Search **"review"[Publication Type]**	2219932
#37	Add	Search **#23 AND #36**	515
#36	Add	Search **#31 NOT #35**	994181
#35	Add	Search **#32 NOT #34**	4302930
#34	Add	Search **#33 AND #32**	16273795
#33	Add	Search **humans[MeSH Terms]**	16273795
#32	Add	Search **animals[MeSH Terms]**	20576725
#31	Add	Search **#24 OR #25 OR #26 OR #27 OR #28 OR #29 OR #30**	1078841
#30	Add	Search **trial[Title]**	163115
#29	Add	Search **randomly[Title/Abstract]**	267055
#28	Add	Search **"clinical trials as topic"[Mesh:noexp]**	178729
#27	Add	Search **placebo[Title/Abstract]**	182580
#26	Add	Search **randomized[Title/Abstract]**	402078
#25	Add	Search **"controlled clinical trial"[Publication Type]**	515779
#24	Add	Search **"randomized controlled trial"[Publication Type]**	429381
#23	Add	Search **#13 AND #22**	1760
#22	Add	Search **#17 OR #21**	4111
#21	Add	Search **#18 OR #19 OR #20**	3055
#20	Add	Search **dutasteride[Title/Abstract]**	671
#19	Add	Search **finasteride[Title/Abstract]**	2265
#18	Add	Search **5-alpha Reductase Inhibitors[Title/Abstract]**	516
#17	Add	Search **#14 OR #15 OR #16**	3041
#16	Add	Search **dutasteride[Supplementary Concept]**	472
#15	Add	Search **finasteride[MeSH Terms]**	1959
#14	Add	Search **5-alpha Reductase Inhibitors[MeSH Terms]**	1759
#13	Add	Search **#1 OR #12**	28335
#12	Add	Search **#10 OR #11**	22160
#11	Add	Search **"BPH"[Title/Abstract]**	9862
#10	Add	Search **#4 AND #9**	19993
#9	Add	Search **#5 OR #6 OR #7 OR #8**	207490
#8	Add	Search **Adenoma[Title/Abstract]**	43703
#7	Add	Search **Adenomas[Title/Abstract]**	31081
#6	Add	Search **Hypertrophy[Title/Abstract]**	75812
#5	Add	Search **Hyperplasia[Title/Abstract]**	79856
#4	Add	Search **#2 OR #3**	170911
#3	Add	Search **Prostate[Title/Abstract]**	151686
#2	Add	Search **Prostatic[Title/Abstract]**	48914
#1	Add	Search **Prostatic Hyperplasia[MeSH Terms]**	19749
