# Supplementary material for: Efficacy and safety of 5 alpha-reductase inhibitor monotherapy in patients with benign prostatic hyperplasia: A meta-analysis
Source: PLoS One. 2018 Oct 3;13(10):e0203479. doi: 10.1371/journal.pone.0203479 (PMC6169865; doi:10.1371/journal.pone.0203479)
Supplement: S3 Text — (PDF) [file pone.0203479.s003.pdf]

#1	MeSH descriptor: [Prostatic Hyperplasia] explode all trees	1536
#2	Prostatic:ab,ti	3310
#3	Prostate:ab,ti	9875
#4	#2 or #3	11202
#5	Hyperplasia:ab,ti	3125
#6	Hypertrophy:ab,ti	2483
#7	Adenomas:ab,ti	919
#8	Adenoma:ab,ti	1169
#9	#5 or #6 or #7 or #8	7102
#10	#4 and #9	2087
#11	"BPH":ab,ti	1188
#12	#10 or #11	2239
#13	#1 or #12	2532
#14	MeSH descriptor: [5-alpha Reductase Inhibitors] explode all trees	202
#15	MeSH descriptor: [Finasteride] explode all trees	319
#16	MeSH descriptor: [Dutasteride] explode all trees	120
#17	#14 or #15 or #16	458
#18	5-alpha Reductase Inhibitors:ab,ti	60
#19	finasteride:ab,ti	484
#20	dutasteride:ab,ti	232
#21	#18 or #19 or #20	705
#22	#17 or #21	744

#23	#13 and #22		369
#24	randomized controlled trial:pt (Word variations have been searched)	s	418842
#25	controlled clinical trial:pt (Word variations have been searched)	s	312498
#26	randomized:ab,ti		371754
#27	placebo:ab,ti		179382
#28	MeSH descriptor: [Clinical Trials as Topic] explode all trees	m	57213
#29	randomly:ab,ti		148564
#30	trial:ti		187569
#31	#24 or #25 or #26 or #27 or #28 or #29 or #30		769921
#32	MeSH descriptor: [Animals] explode all trees	m	7971
#33	MeSH descriptor: [Humans] explode all trees	m	1457
#34	#33 and #32		1457
#35	#32 not #34		6514
#36	#31 not #35		763538
#37	#23 and #36		327
#38	review:pt (Word variations have been searched)	s	6472
#39	MeSH descriptor: [Review Literature as Topic] explode all trees	m	163
#40	#38 or #39		6633
#41	#37 not #40<br>Publication Year from 2015 to 2017		23
