# Supplementary material for: Efficacy and safety of 5 alpha-reductase inhibitor monotherapy in patients with benign prostatic hyperplasia: A meta-analysis
Source: PLoS One. 2018 Oct 3;13(10):e0203479. doi: 10.1371/journal.pone.0203479 (PMC6169865; doi:10.1371/journal.pone.0203479)
Supplement: S1 Table — (DOCX) [file pone.0203479.s004.docx]

| eTable 1. Methodological qualities of included studies | | | | | | |
| --- | --- | --- | --- | --- | --- | --- |
| Study | Random sequence generation | Allocation concealment | Blinding of participants  and personnel | Blinding of outcome assessment | Incomplete outcome data | Selective reporting |
| Beisland_1992 | Low risk (described 'randomized') | Unclear | Low risk (double-blind) | Low risk (double-blind) | Low risk (described analysis using and all-patients-treated approach estimates, drop out rate due to adverse event; Treatment F5mg6.3%, Placebo1.1%) | Low risk  (The study protocol was reported in the pre-specified way) |
| Gormley_1992 | Low risk (described 'randomized') | Unclear | Low risk (double-blind) | Low risk (double-blind) | Low risk (described ITT analysis, drop out rate due to adverse event; Treatment F1mg5%, F5mg5%, Placebo6%) | Low risk  (The study protocol was reported in the pre-specified way) |
| kirby_1992 | Low risk (described 'randomized') | Unclear | Low risk (double-blind) | Low risk (double-blind) | Unclear (drop out rate due to adverse event; Treatment 2.4%) | Low risk  (The study protocol was reported in the pre-specified way) |
| Stoner_1992 | Unclear | Unclear | Low risk (double-blind) | Low risk (double-blind) | Low risk (nearly assumed ITT analysis using whole sample set, drop out rate due to adverse event total 5) | Low risk  (The study protocol was reported in the pre-specified way) |
| Tammela_1993 | Low risk (described 'randomized') | Unclear | Low risk (double-blind) | Low risk (double-blind) | Low risk (nearly assumed ITT analysis using whole sample set, no mention of drop out rate due to adverse event) | Low risk  (The study protocol was reported in the pre-specified way) |
| Tempany_1993 | Low risk (described 'randomized') | Unclear | High risk (no mention of double-blind) | Low risk (blind) | Low risk (nearly assumed ITT analysis using whole sample set, no mention of drop out rate due to adverse event) | Low risk  (The study protocol was reported in the pre-specified way) |
| The finasteride study group _1993 | Low risk (described 'randomized') | Unclear | Low risk (double-blind) | Low risk (double-blind) | Low risk (nearly assumed ITT analysis using whole sample set, drop out rate due to adverse event F5mg;0.4%) | Low risk  (The study protocol was reported in the pre-specified way) |
| Stoner_1994 | Low risk (described 'randomized') | Unclear | Low risk (double-blind) | Low risk (double-blind) | Low risk (nearly assumed ITT analysis using whole sample set, no mention of drop out rate due to adverse event) | Low risk  (The study protocol was reported in the pre-specified way) |
| Andersen_1995 | Low risk (described 'randomized') | Unclear | Low risk (double-blind) | Low risk (double-blind) | Unclear (drop out rate due to adverse event; Treatment 8.5%, Placebo 11%) | Low risk  (The study protocol was reported in the pre-specified way) |
| Tammela_1995 | Low risk (described 'randomized') | Unclear | Low risk (double-blind) | Low risk (double-blind) | Low risk (nearly assumed ITT analysis using whole sample set, no mention of drop out rate due to adverse event) | Low risk  (The study protocol was reported in the pre-specified way) |
| Yu_1995 | Low risk (described 'randomized') | Unclear | Low risk (double-blind) | Low risk (double-blind) | Unclear (drop out rate due to adverse event_ no mention of drop out rate due to adverse event) | Low risk  (The study protocol was reported in the pre-specified way) |
| Lepor_1996 | Low risk (described 'randomized') | Low risk (randomly assigned by a central computer) | Low risk (double-blind) | Low risk (double-blind) | Low risk (described ITT analysis, no mention of drop out rate due to adverse event) | Low risk  (The study protocol was reported in the pre-specified way) |
| Nickel_1996 | Low risk (described 'randomized') | Low risk (randomly assigned according to a computer-generated scheduler) | Low risk (double-blind) | Low risk (double-blind) | Low risk (described ITT analysis, drop out rate due to adverse event;  Treatment 9%, Placebo 13%) | Low risk  (The study protocol was reported in the pre-specified way) |
| Habib_1997 | Low risk (described 'randomized') | Unclear | Low risk (double-blind) | Low risk (double-blind) | Low risk (nearly assumed ITT analysis using whole sample set, no mention of drop out rate due to adverse event) | Low risk  (The study protocol was reported in the pre-specified way) |
| Lepor_1998 | Low risk (described 'randomized') | Low risk (randomized by a central computer in equal proportions) | Low risk (double-blind) | Low risk (double-blind) | Low risk (described ITT analysis, no mention of drop out rate due to adverse event) | High risk (there is no mention of  52 weeks PSA) |
| Marberger_1998 | Low risk (described 'randomized') | Low risk (1:1 randomization) | Low risk (double-blind) | Low risk (double-blind) | Unclear (baseline N F; 1450, P;1452, PV measurement N F;776, P;800_dropout rate due to adverse events F5mg 7.4%, Placebo 9.4%) | Low risk  (The study protocol was reported in the pre-specified way) |
| McConnell_1998 | Low risk (described 'randomized') | Low risk (computer generated schedule, without stratification according to site) | Low risk (double-blind) | Low risk (double-blind) | Low risk  (described ITT analysis, drop out rate due to adverse event; F5mg 11.5%, Placebo 10.9%) | Low risk  (The study protocol was reported in the pre-specified way) |
| Pannek_1998 | Low risk (described 'randomized') | Low risk (2:1 randomization) | Low risk (double-blind) | Low risk (double-blind) | Unclear (described "There were no patients lost to follow up or dropped from the study" but measurement number of patient is different) | Low risk  (The study protocol was reported in the pre-specified way) |
| Abrams_1999 | Low risk (described 'randomized') | Low risk (2:1 randomization) | Low risk (double-blind) | Low risk (double-blind) | Unclear (no mention of drop out rate due to adverse event and measurement number of patient is different) | Low risk  (The study protocol was reported in the pre-specified way) |
| Lukkarinen_1999 | Low risk (described 'randomized') | Unclear | Low risk (double-blind) | Low risk (double-blind) | Unclear (no mention of drop out rate due to adverse event and measurement number of patient is different) | Low risk  (The study protocol was reported in the pre-specified way) |
| Schafer_1999 | Low risk (described 'randomized') | Unclear | Low risk (double-blind) | Low risk (double-blind) | Unclear (no mention of drop out rate due to adverse event and measurement number of patient is different baseline;81, after 12months; 69 ) | Low risk  (The study protocol was reported in the pre-specified way) |
| Feneley_2000 | Low risk (described 'randomized') | Low risk (2:1 randomization) | High risk (there is no mention of double-blind) | Low risk (blind) | Unclear | Low risk  (The study protocol was reported in the pre-specified way) |
| Isotalo_2001 | Low risk (described 'randomized') | Unclear | Low risk (double-blind) | Low risk (double-blind) | Low risk (described ITT analysis, drop out rate of adverse event.) | Unclear (There were no statistically significant changes either within or between the two groups for the IPSS or quality of- life score) |
| Espana_2002 | Low risk (described 'randomized') | Low risk (3:1 randomization) | High risk (there is no mention of double-blind) | Low risk (blind) | Unclear (no mention of drop out rate due to adverse event) | Low risk  (The study protocol was reported in the pre-specified way) |
| Haggstrom_2002 | Low risk (described 'randomized') | Unclear | High risk (there is no mention of double-blind) | Low risk (blind) | Low risk (nearly assumed ITT analysis using whole sample set) | Low risk  (The study protocol was reported in the pre-specified way) |
| Roehrborn_2002 | Low risk (described 'randomized') | Unclear | Low risk (double-blind) | Low risk (double-blind) | Low risk (described ITT analysis, drop out rate due to adverse events:  Treatment 8.9%, Placebo 8.8%) | Low risk  (The study protocol was reported in the pre-specified way) |
| Kirby_2003 | Low risk (described 'randomized') | Unclear | Low risk (double-blind) | Low risk (double-blind) | Low risk (described ITT analysis, drop out rate due to adverse events:  Treatment 12.9%, Placebo 11.1%) | Low risk  (The study protocol was reported in the pre-specified way) |
| McConnell_2003 | Low risk (described 'randomized') | Unclear | Low risk (double-blind) | Low risk (double-blind) | Low risk (described ITT analysis, drop out rate due to adverse events;  Treatment 24%) | Low risk  (The study protocol was reported in the pre-specified way) |
| Roehrborn_2004 | Low risk (described 'randomized') | Low risk (blinded to treatment allocation) | Low risk (double-blind) | Low risk (double-blind) | Low risk (described ITT analysis, no mention of drop out rate due to adverse event) | Low risk  (The study protocol was reported in the pre-specified way) |
| Crawford_2006 | Low risk (described 'randomized') | Unclear | Low risk (double-blind) | Low risk (double-blind) | Low risk (described ITT analysis, no mention of drop out rate due to adverse event) | Low risk  (The study protocol was reported in the pre-specified way) |
| Gittelman_2006 | Low risk (described 'randomized') | Unclear | Low risk (double-blind) | Low risk (double-blind) | Low risk (described ITT analysis, no mention of drop out rate due to adverse event) | Low risk  (The study protocol was reported in the pre-specified way) |
| Kaplan_2006 | Low risk (described 'randomized') | Unclear | Unclear | Unclear | Low risk (described ITT analysis, no mention of drop out rate due to adverse event) | Low risk  (The study protocol was reported in the pre-specified way) |
| Kaplan_2008 | Low risk (described 'randomized') | Unclear | High risk (there is no mention of double-blind) | Low risk (blind) | Low risk (described ITT analysis, no mention of drop out rate due to adverse event) | High risk (there is no mention of AUA-SS) |
| Bepple_2009 | Low risk (described 'randomized') | Unclear | Low risk (double-blind) | Low risk (double-blind) | Low risk (described ITT analysis, no mention of drop out rate due to adverse event) | Low risk  (The study protocol was reported in the pre-specified way) |
| Tsukamoto_2009 | Low risk (described 'randomized') | Unclear | Low risk (double-blind) | Low risk (double-blind) | Low risk (nearly assumed ITT analysis using whole sample set, drop out rate due to adverse event:  Treatment_D0.05mg 4.2%, D0.5mg 4.1%, D2.5mg 10%, Placebo 12.5%) | Low risk  (The study protocol was reported in the pre-specified way) |
| Tsukamoto_2009 | Low risk (described 'randomized') | Unclear | Low risk (double-blind) | Low risk (double-blind) | Low risk (nearly assumed ITT analysis using whole sample set, drop out rate due to adverse event:  Treatment 7.7%,Placebo 4.8%) | Low risk  (The study protocol was reported in the pre-specified way) |
| Tsukamoto_2010 | Low risk (described 'randomized') | Unclear | High risk (there is no mention of double-blind) | Low risk (blind) | Unclear | Unclear |
| Kaplan_2011 | Low risk (described 'randomized') | Low risk (subject were randomly  assigned 1:1:1:1 ratio) | High risk (there is no mention of double-blind) | Low risk (blind) | Low risk (described ITT analysis, no mention of drop out rate due to adverse event) | Low risk  (The study protocol was reported in the pre-specified way) |
| Roehrborn_2011 | Low risk (described 'randomized') | Unclear | Low risk (double-blind) | Low risk (double-blind) | Unclear | Low risk  (The study protocol was reported in the pre-specified way) |
| Yanqun_2012 | Low risk (described 'randomized') | Low risk (subject were randomly assigned 1:1 ratio and maintained by the Glaxo smith Kline Randall system) | Low risk (double-blind) | Low risk (double-blind) | Low risk (described ITT analysis, drop out rate due to adverse event:  Treatment 2.3%, Placebo 0%) | Low risk  (The study protocol was reported in the pre-specified way) |
| Kacker_2015 | Low risk (described 'randomized') | Low risk (subject were randomly  assigned 1:1 ratio) | Low risk (double-blind) | Low risk (double-blind) | Low risk (nearly assumed ITT analysis using whole sample set. No participants withdrew from the study due to side effects from treatment or placebo) | Low risk  (The study protocol was reported in the pre-specified way) |
| Qian_2015 | Low risk (described 'randomized') | Low risk (computer generated randomization schedule) | Low risk (double-blind) | Low risk (double-blind) | Low risk (nearly assumed ITT analysis using whole sample set, no mention of drop out rate due to adverse even ) | Low risk  (The study protocol was reported in the pre-specified way) |
| ITT: intent-to treat. | | | | | | |
