# Supplementary material for: Efficacy and safety of 5 alpha-reductase inhibitor monotherapy in patients with benign prostatic hyperplasia: A meta-analysis
Source: PLoS One. 2018 Oct 3;13(10):e0203479. doi: 10.1371/journal.pone.0203479 (PMC6169865; doi:10.1371/journal.pone.0203479)
Supplement: S2 Table — (DOCX) [file pone.0203479.s005.docx]

| eTable 2. Meta-regression analysis for ratio of means meta-analysis of the efficacy of placebo group | | | | | |
| --- | --- | --- | --- | --- | --- |
| Dependent variable | Independent Variable | Univariable | | Multivariable | |
|  |  | B (95% CI) | p-value | B (95% CI) | p-value |
| Log (ROM of PSA) | Publication year | -0.007 (-0.027, 0.013) | 0.508 | -0.005 (-0.032, 0.021) | 0.694 |
|  | F/U duration (month) | -0.003 (-0.012, 0.006) | 0.532 | -0.003 (-0.013, 0.007) | 0.569 |
| Log (ROM of PV) | Publication year | -0.006 (-0.025, 0.013) | 0.525 | -0.021 (-0.026, -0.015) | <0.001 |
|  | F/U duration (month) | 0.008 (0.003, 0.012) | 0.002 | 0.012 (0.01, 0.013) | <0.001 |
| Log (ROM of PVR) | Publication year | -0.129 (-0.167, -0.091) | <0.001 | -0.139 (-0.159, -0.119) | <0.001 |
|  | F/U duration (month) | -0.068 (-1.669, 1.532) | 0.933 | 0.344 (-0.067, 0.756) | 0.101 |
| Log (ROM of IPSS) | Publication year | -0.031 (-0.05, -0.012) | 0.001 | -0.033 (-0.048, -0.017) | <0.001 |
|  | F/U duration (month) | 0.005 (-0.003, 0.013) | 0.186 | 0.006 (0.001, 0.013) | 0.040 |
| Log (ROM of Qmax) | Publication year | 0.026 (0.017, 0.035) | <0.001 | 0.029 (0.019, 0.039) | <0.001 |
|  | F/U duration (month) | -0.002 (-0.006, 0.002) | 0.365 | -0.004 (-0.007, -0.001) | 0.020 |
| ROM, ratio of means; PSA, prostate specific antigen; PV, prostate volume; PVR, post voided residual volume; IPSS, International Prostate Symptom Score; Qmax, maximal urinary flow rate; F/U, follow up. | | | | | |
