# Supplementary figures and images for: Efficacy and safety of 5 alpha-reductase inhibitor monotherapy in patients with benign prostatic hyperplasia: A meta-analysis
Source: PLoS One. 2018 Oct 3;13(10):e0203479. doi: 10.1371/journal.pone.0203479 (PMC6169865; doi:10.1371/journal.pone.0203479)

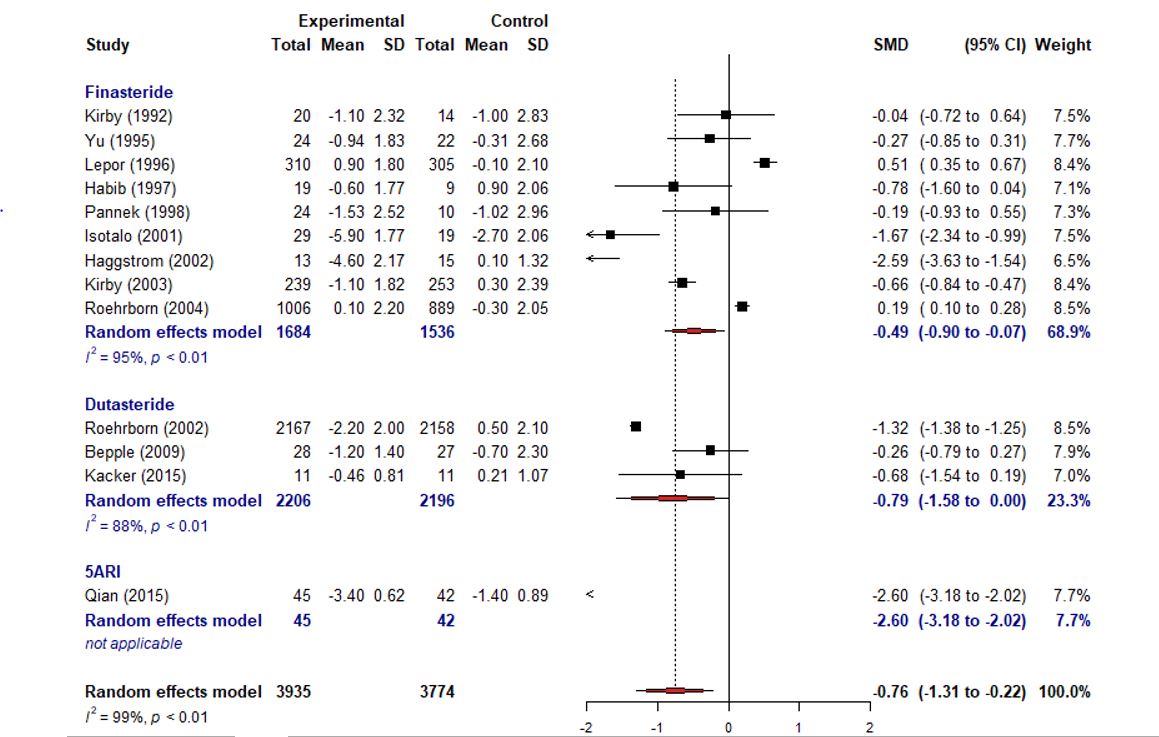

Supplement: S1 Fig — (JPG) [file pone.0203479.s006.JPG]

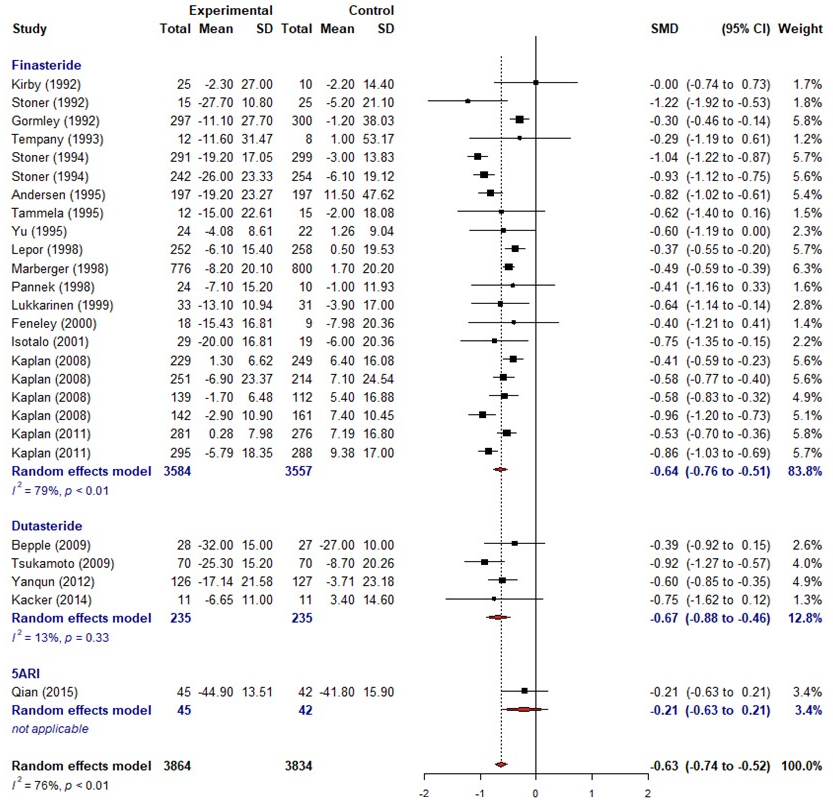

Supplement: S2 Fig — (TIF) [file pone.0203479.s007.tif]

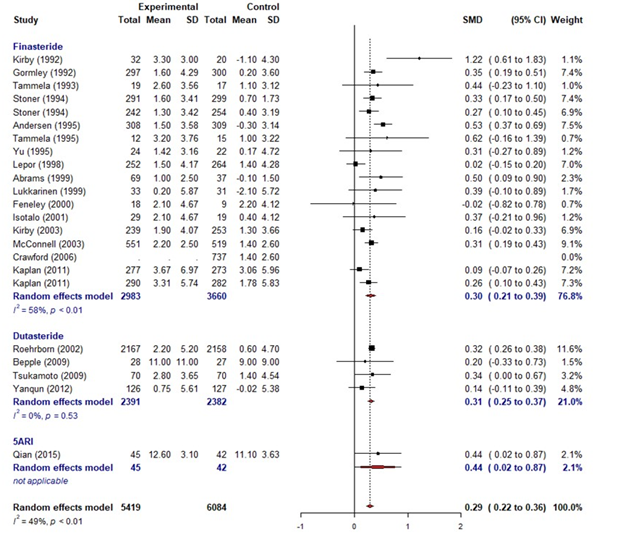

Supplement: S3 Fig — (TIF) [file pone.0203479.s008.tif]

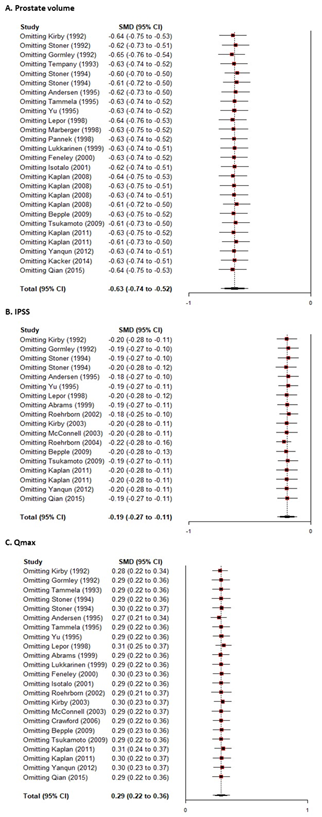

Supplement: S4 Fig — (TIF) [file pone.0203479.s009.tif]

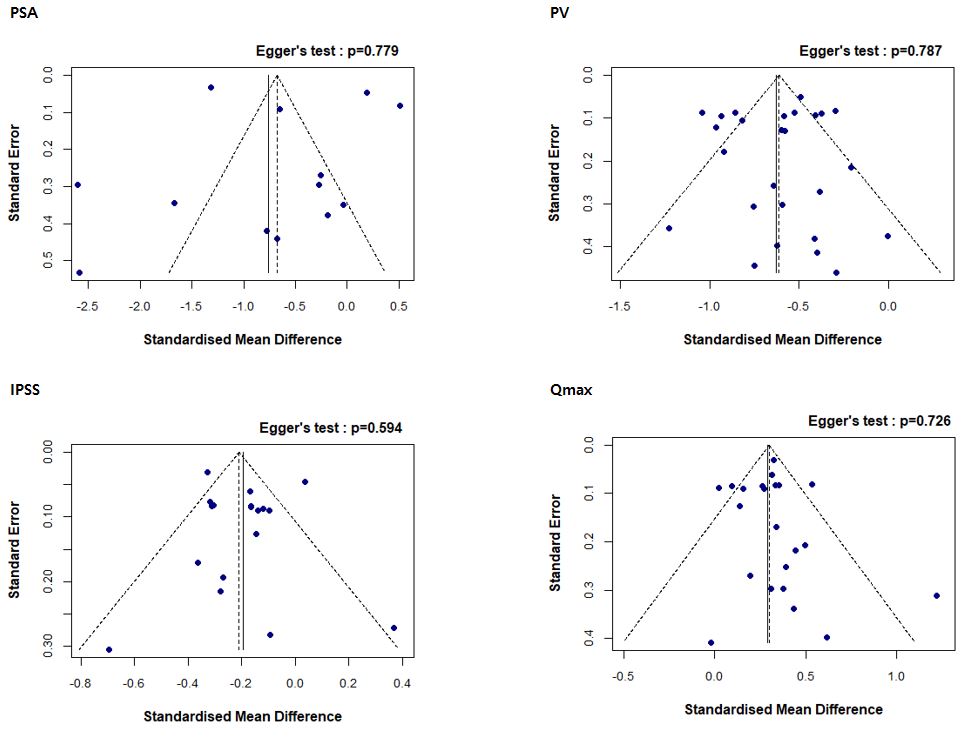

Supplement: S5 Fig — (JPG) [file pone.0203479.s010.JPG]
